# Supplementary material for: A SAM-key domain required for enzymatic activity of the Fun30 nucleosome remodeler
Source: Life Sci Alliance. 2023 Jul 19;6(9):e202201790. doi: 10.26508/lsa.202201790 (PMC10355287; doi:10.26508/lsa.202201790)
Supplement: Supplementary file 1 [file LSA-2022-01790_SdataF2_F3_F6.pdf]

Figure 2

Figure 2A

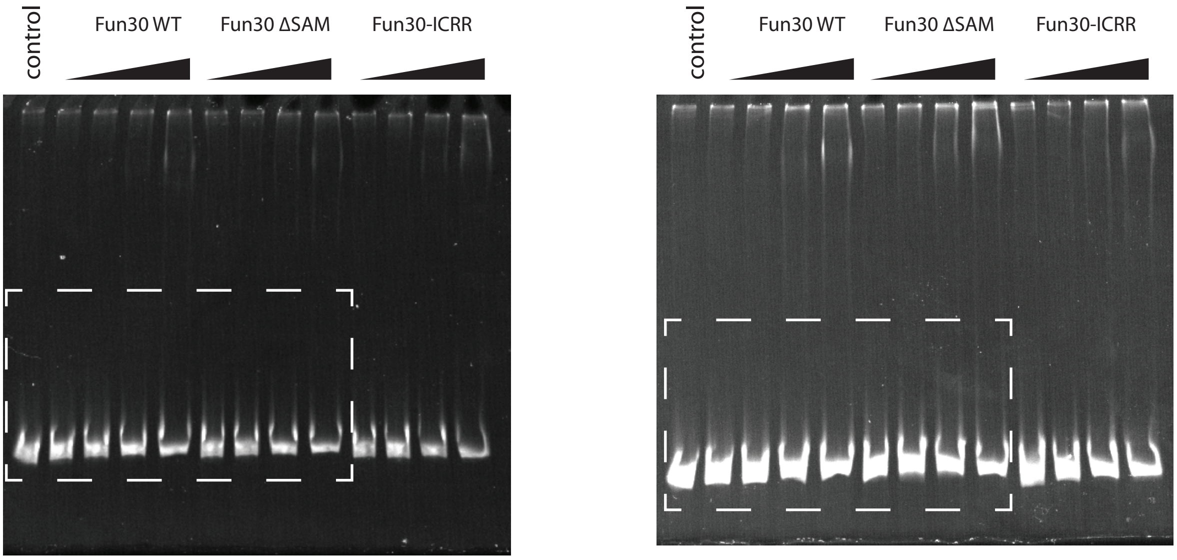

Figure 2B

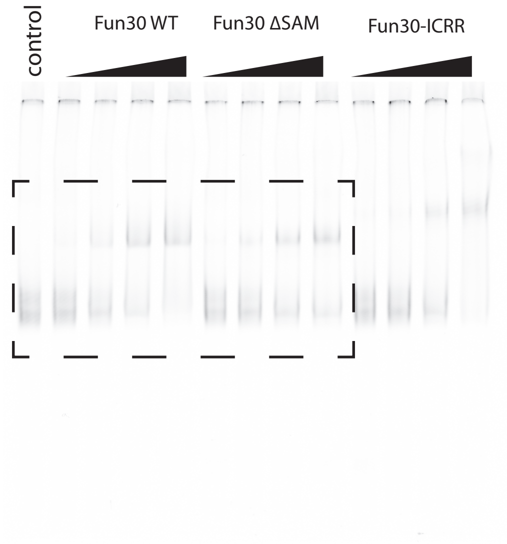

Figure 2C

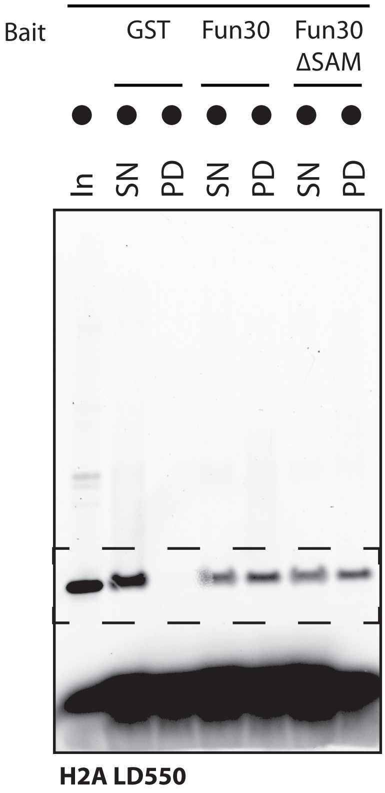

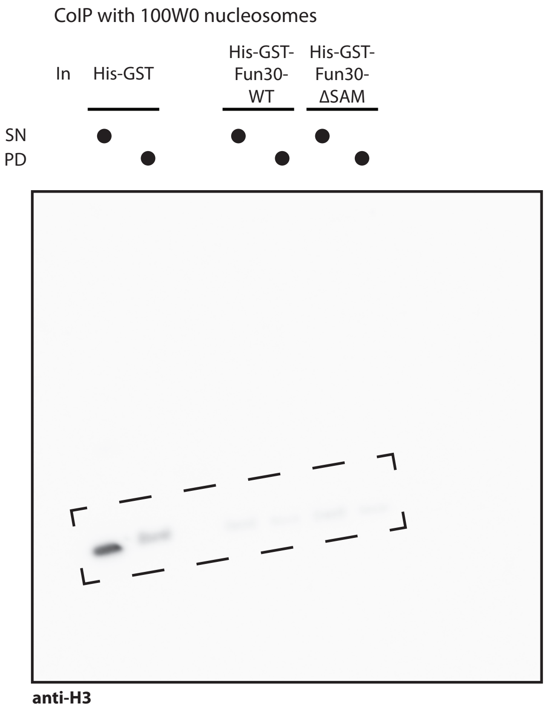

Figure 3

Figure 3B

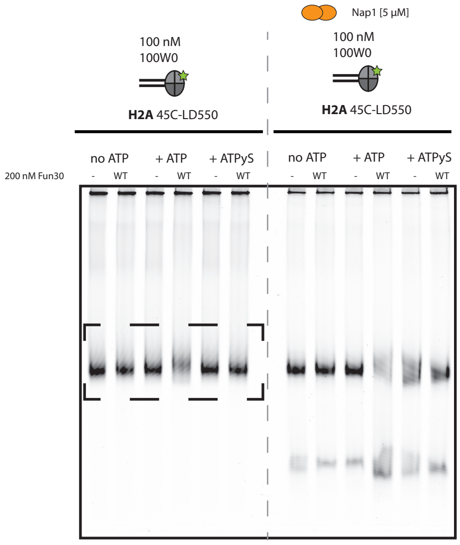

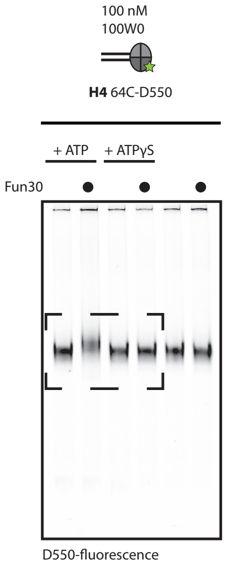

Figure 3C

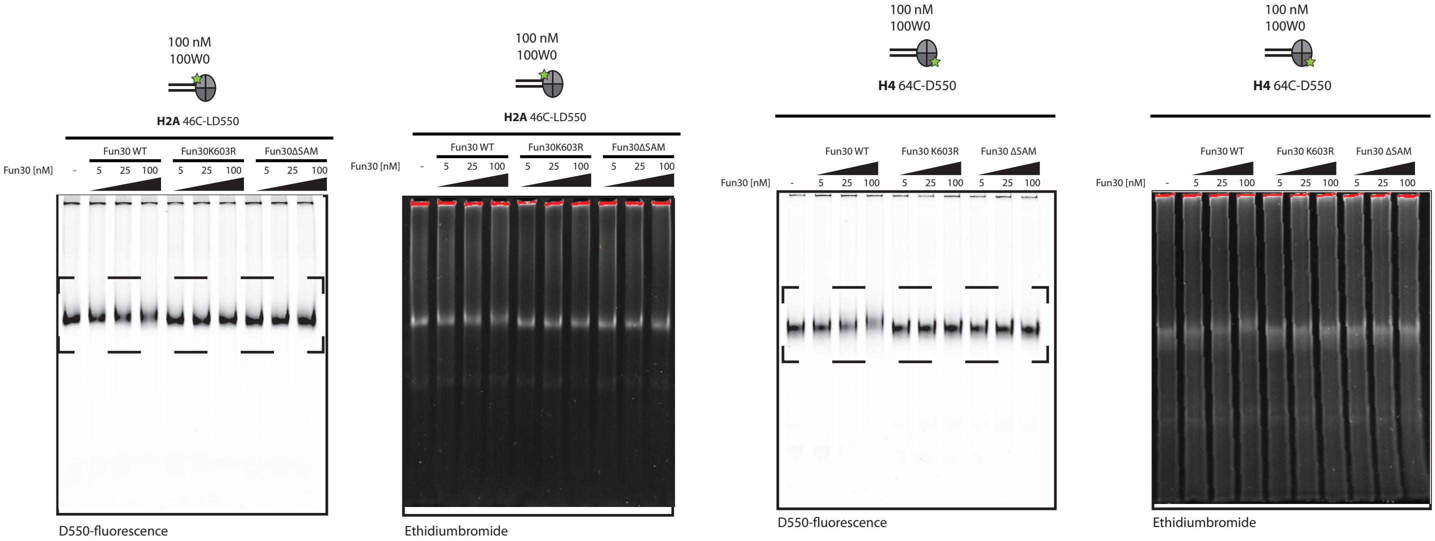

Figure 3E

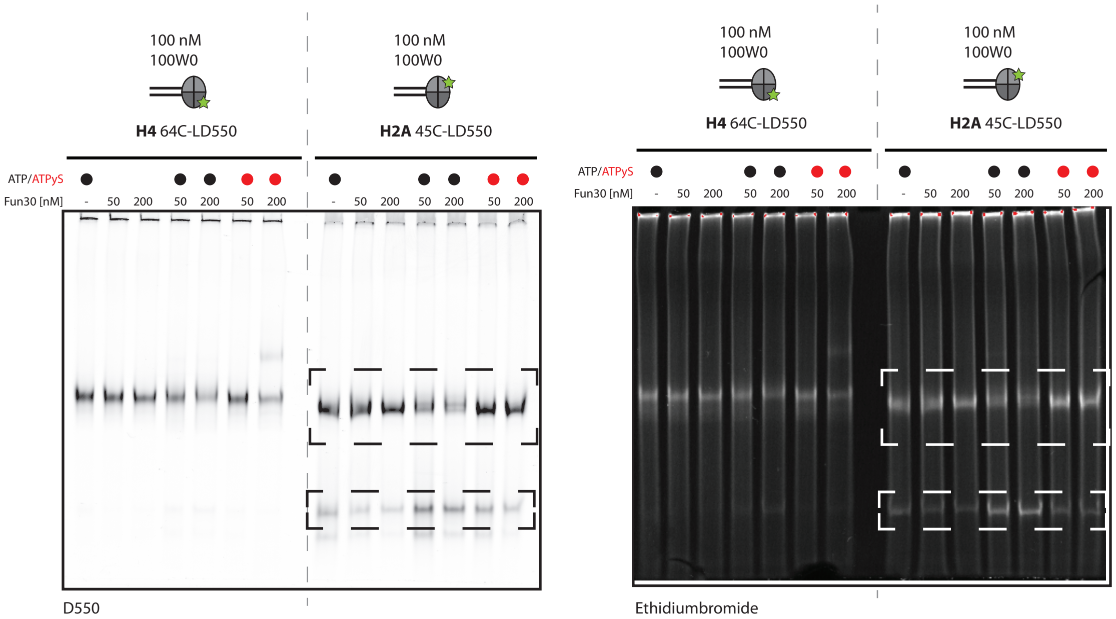

Figure 3F

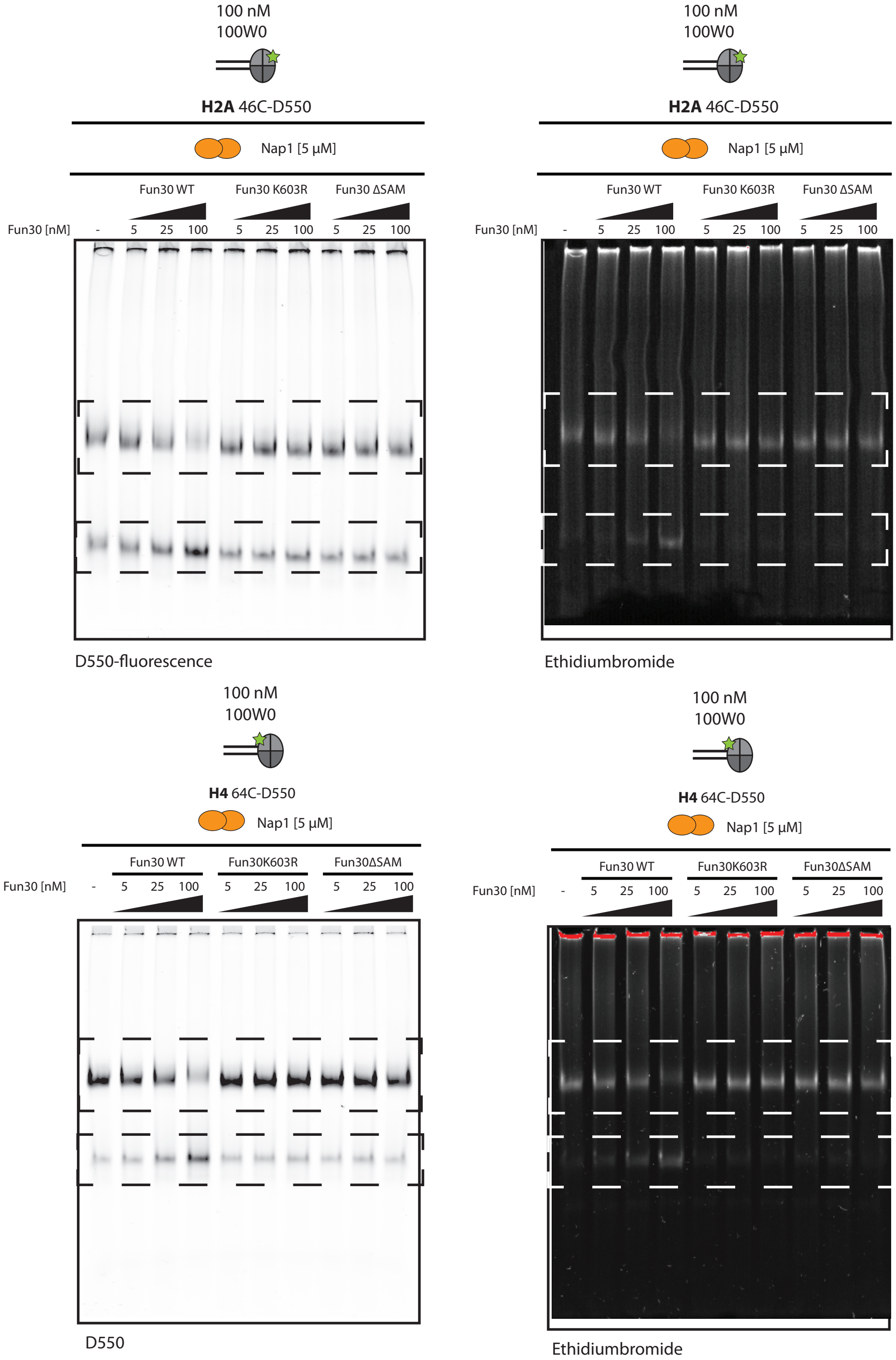

Figure 3K

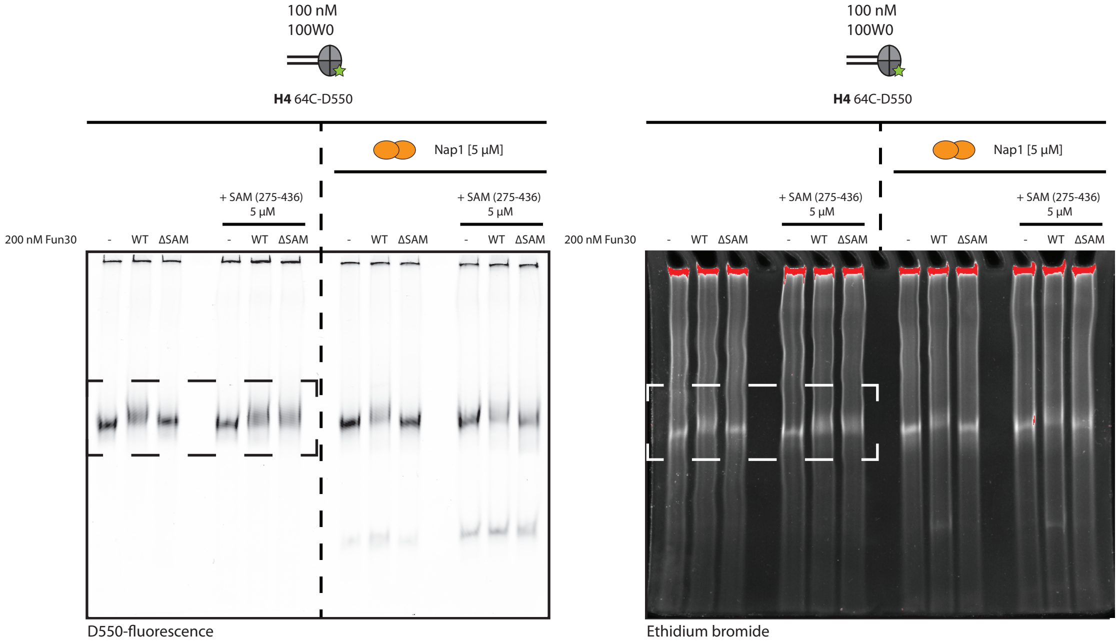

Figure 6

Figure 6A

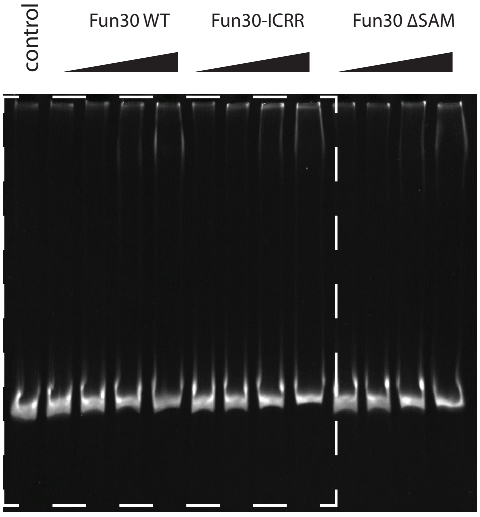

Figure 6C

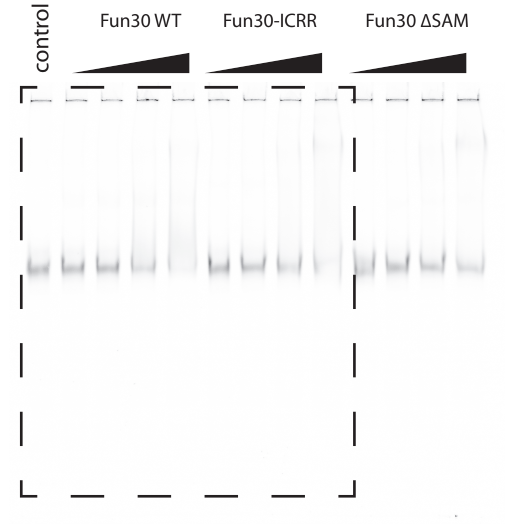

Figure 6E & Figure 6F

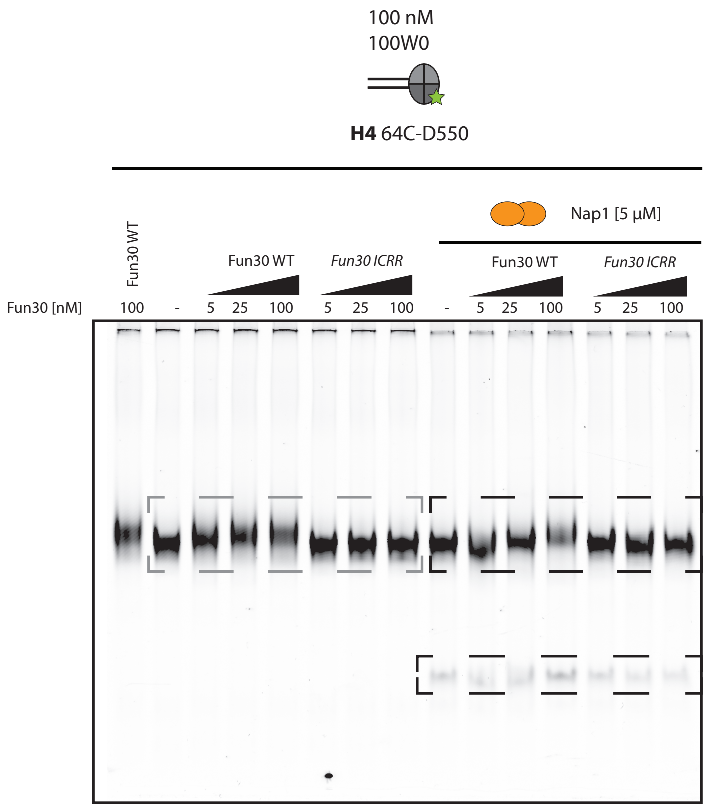

D550-fluorescence

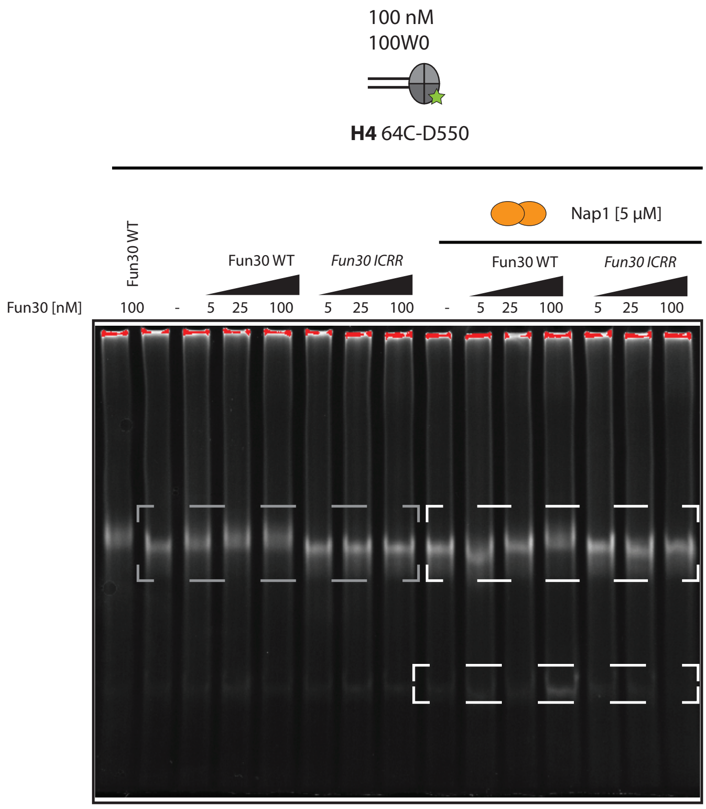

Ethidiumbromide
